# Quality of Life Evaluation of Postsurgical Mandibular Fracture Patients with Oral Health Impact Profile 14 and General Oral Health Assessment Index Parameters

**DOI:** 10.1055/s-0043-1761450

**Published:** 2023-03-28

**Authors:** Ardian Jayakusuma Amran, Andra Rizqiawan, Indra Mulyawan, Okky Prasetio, Eko Wicaksono Subagio, Mohammad Zeshaan Rahman

**Affiliations:** 1 Postgraduate Program, Oral and Maxillofacial Surgery, Faculty of Dental Medicine, Universitas Airlangga, Surabaya, Indonesia; 2 Department of Oral and Maxillofacial Surgery, Faculty of Dental Medicine, Universitas Muslim Indonesia, Indonesia; 3 Department of Oral and Maxillofacial Surgery, Faculty of Dental Medicine, Universitas Airlangga, Surabaya, Indonesia; 4 Department of Oral and Maxillofacial Surgery Dental Hospital, Universitas Airlangga, Surabaya, Indonesia; 5 Department of Oral and Maxillofacial Surgery, M. Soewandhie Hospital, Surabaya, Indonesia; 6 Department Oral and Maxillofacial Surgery, Pioneer Dental College and Hospital, Dhaka, Bangladesh

**Keywords:** mandibular fracture, quality of life, OHIP 14, GOHAI, oral health

## Abstract

**Objective** Mandibular fracture is the most common maxillofacial fracture accompanied by complaints of malocclusion and pain. This causes a decrease in the quality of life. Mandibular fracture management can be done with open reduction and internal fixation or intermaxillary fixation. The Oral Health Impact Profile (OHIP 14) and the General Oral Health Assessment Index (GOHAI) were used to evaluate the quality of life after surgical treatment based on the distribution of age, sex, type of neglect, and surgical management.

**Materials and Methods** This research is an analytic study with an analytical observational method with total sampling. The total sample used was 15 patients during the 2006 to 2020 period. The results of this study were scored, and then, the data were processed using the eta test.

**Results** The results of the study based on the OHIP 14 parameters showed the results of each distribution, namely, age: *p* = 0.154, gender: *p* = 0.080, neglected type: *p* = 0.080, and management: *p* = 0.419. Meanwhile, the GOHAI parameters showed the results of each distribution, namely, age: *p* = 0.105, gender: *p* = 0.356, neglected type: *p* = 0.356, and management *p* = 0.286. The results of this distribution showed that there was no significant difference between patients' quality of life based on age, sex, neglected type, and treatment using both OHIP 14 and GOHAI parameters.

**Conclusions** The results obtained in this study using characteristics of age, gender, type of fracture, type of neglect, and management did not have a significant effect on the level of patient satisfaction after surgery, using both OHIP 14 and GOHAI questionnaires.

## Introduction

The incidence of maxillofacial trauma is increasing every year, especially in big cities. This is in line with the development of lifestyles and increasingly modern means of transportation. Maxillofacial trauma is mostly caused by traffic accidents. Mandibular fracture is the most common fracture of the maxillofacial region, with complaints of malocclusion and pain in the fracture region accompanied by loose or detached teeth, soft tissue damage such as edema, contusions, abrasions, lacerations, and avulsions. 1 These complaints reduce the patient's quality of life.

The incidence of fractures in the mandible is generally easy to diagnose because most of the patients complain of malocclusion and pain in the fracture region. The mandible will usually be fractured in two places, namely at the site of impact and the contralateral area of the impact site. Fractures in the contralateral region usually involve the condyle of the mandible or the angle of the mandible on the contralateral side or are called indirect fractures. 2 Fractures of the mandible can also cause malocclusion, inferior alveolar nerve paresthesias, and ankylosis. In addition, it can cause infection and osteomyelitis if treatment is not taken immediately. 3

Mandibular fracture treatment can be done with closed reduction, open reduction, or a combination of both to restore normal occlusion in patients with mandibular fractures. 4 5 6 7

The quality of life of patients can be measured using parameters as instruments, several parameters can be used to measure the quality of life related to oral health. One of the instruments that are often used to measure the quality of life-related to the oral cavity is the Oral Health Impact Profile (OHIP 14). 8 9 10 The OHIP is a reliable measure for oral health-related quality of life (OHRQoL). Of the seven dimensions of OHRQoL, each has two items to form 14 questions (OHIP-14). The seven dimensions measured were functional limitations (D1), pain (D2), psychological discomfort (D3), physical (D4) and psychological (D5) disabilities, social disabilities (D6), and handicaps (D7). Responses to this scale are based on a Likert format with a five-point ordinal scale (never [0], almost never [1], sometimes [2], quite often [3], and very often [4]). The total OHIP-14 score range is 0 to 56, where the higher the score, the worse the quality of life. 11 Another instrument commonly used is the Geriatric/General Oral Health Assessment Index (GOHAI), this instrument was developed for the assessment of the quality of life related to oral health. 12 13 14 15 16

The level of quality of life after surgery is very important and can be a measure of patient satisfaction level after the procedure. The aim of this study is to measure patients' level of quality of life with mandibular fractures after surgery using the OHIP 14 and GOHAI measurement instruments. The hypothesis of this study is that patients who have undergone surgery have a good quality of life.

## Materials and Methods

This research has been approved by the ethics committee of the Faculty of Dental Medicine, Universitas Airlangga, Surabaya No. 467/HRECC. FODM/X/2020. The study was conducted by the retrospective analytic observational method with inclusion criteria being patients diagnosed with a mandibular fracture who had undergone surgery whether it is with close reduction and open reduction in the Oral and Maxillofacial Surgery Division and those willing to provide informed consent. Meanwhile, mandibular fracture patients who did not have complete data, had communication disorders, did not receive surgical treatment, and were not willing to provide informed consent were excluded from the study sample.

**Fig. 1:**
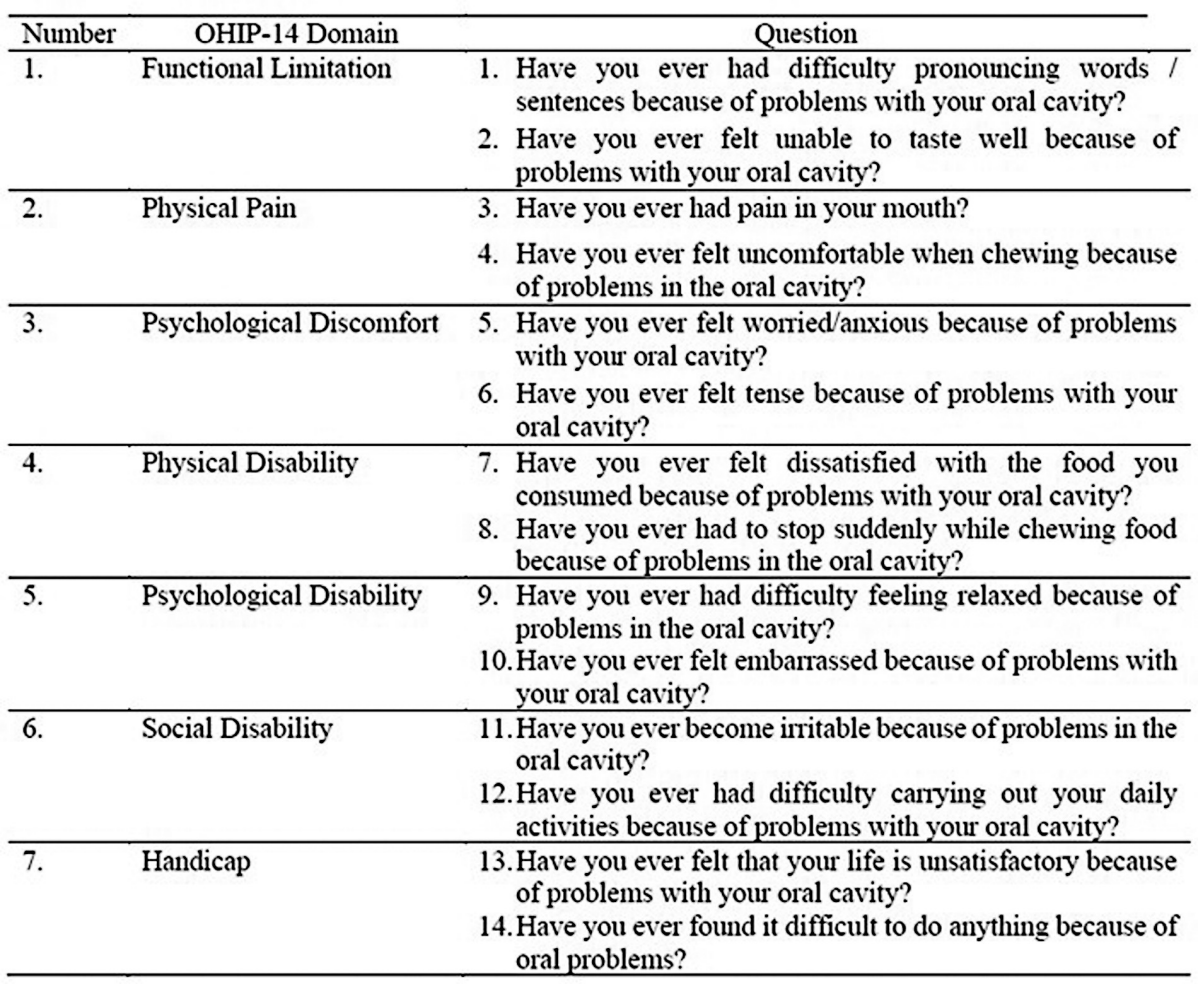
OHIP-14 Questionnaire. Score 0 to 4 (0: Never, 1: Seldom, 2: Sometimes, 3: Often, 4: Very Often). High quality of life (<19), medium quality of life (19–37), and low quality of life (38–56).

**Fig. 2:**
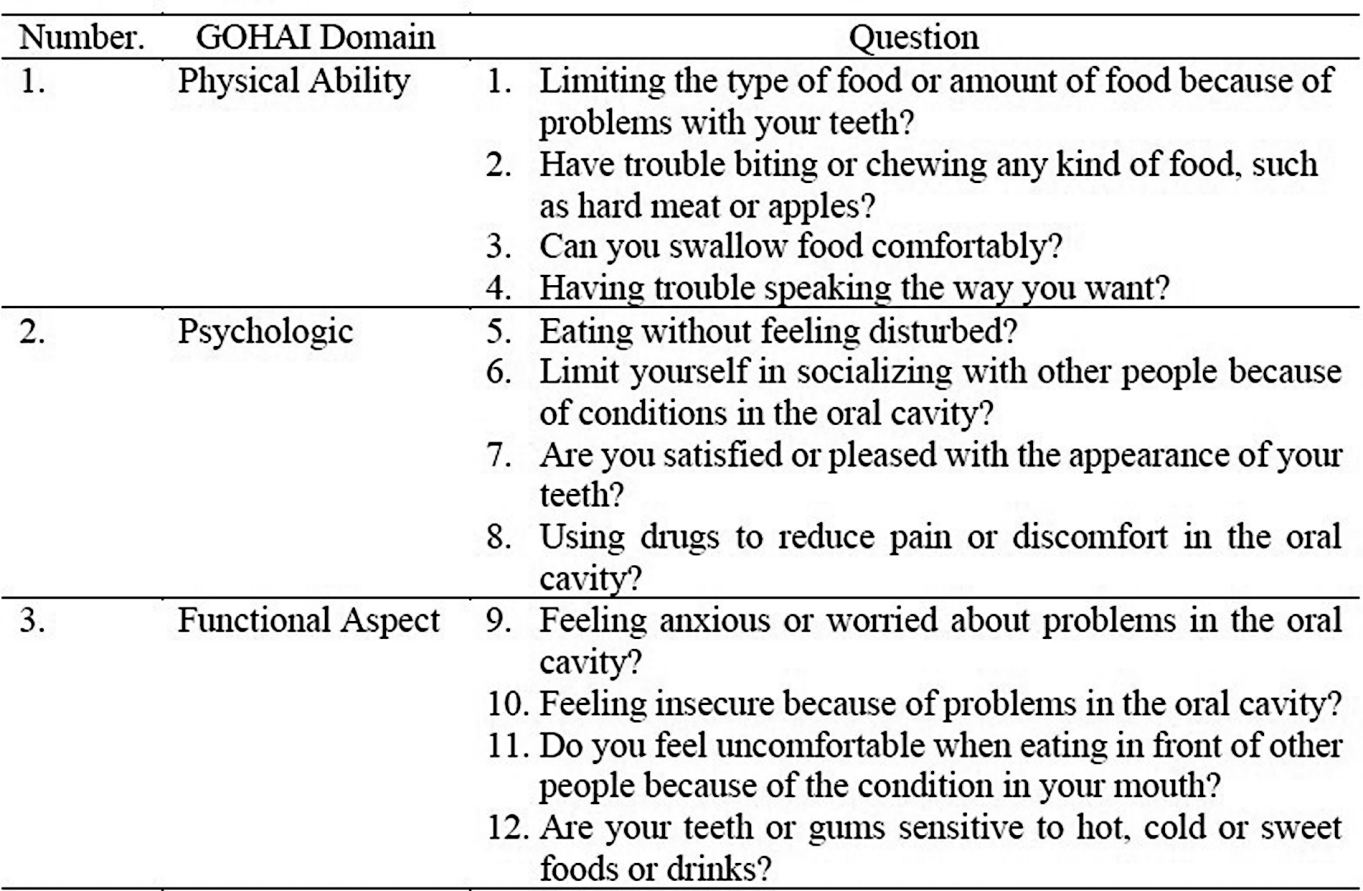
GOHAI Questionnaire. Score 1 to 3 (1: always, 2: sometimes, 3: never). High quality of life (34–36), medium quality of life (31–33), and low quality of life (<30).

The patient data studied were age, gender, telephone number, type of mandibular fracture, and treatment given from medical records. The sample of this study was the entire population (total sampling) due to the small number of subjects (less than 100), namely 15 people with a diagnosis of mandibular fracture who had undergone surgery in the Oral and Maxillofacial Surgery Division. The sample was then given OHIP 14 ( Fig. 1 ) and GOHAI ( Fig. 2 ) which had been used in several previous studies.

## Data Analysis

Filling out the questionnaire was done by telephone, considering the pandemic coronavirus disease 2019 conditions that made it impossible to meet directly with patients. The results of filling out the questionnaire were scored and tabulated. Then, the relationship between the quality of life variables OHIP-14 and GOHAI with the sample character was analyzed using the eta test using SPSS program version 26.

## Results

In the study, the total number of patients with mandibular fractures was 15 patients who had been treated and were willing to participate in this study. Based on gender data, nine people were male (60%) and six were female (40%). Meanwhile, based on the age range, at the age of 18 to 40 years, there were 13 people (86.67%) and at the age of 41 to 65 years, there were 2 people (13.33%). Based on mandibular fracture, there were four patients with condylar fracture (26.7%), condyle fracture with parasymphysis fracture, and condyle fracture with symphysis fracture each having as many as two patients (13.33%) while for other types of fracture one patient each (6.67%).

### Response Distribution Based on Oral Health Impact Profile 14

Based on the OHIP 14 response to age, at the age of 18 to 40 years, there were 13 patients, of which 11 patients were in a high OHIP 14 category (84%), 1 patient was in a moderate, and 1 patient was in a low category (7.7%). Meanwhile, in the age range of 41 to 65 years, there were two patients, of which two patients were in a high OHIP 14 category (100%). See Table 1 .

**Table 1:** Distribution of OHIP 14 responses to age

Variable		OHIP 14 category			Total
		Low	Moderate	High	
Age	18–40 y	1(7.7%)	1(7.7%)	11(84.6%)	13
	41–65 y	0(0%)	0(0%)	2(100%)	2
Total		1 (6.7%)	1(6.7%)	13(86.7%)	15(100%)

Abbreviation: OHIP, Oral Health Impact Profile.

*p* -Value = 0.145.

In male patients, there were nine patients, of which eight patients were in a high OHIP 14 category (88.9%), one patient was in a moderate category (11.1%), and there were no patients in the low category. Meanwhile, for female patients, there were six patients, of which five patients were in a high OHIP 14 category (83.3%), no patient was found in the medium category, and one patient was in a low category (16.7%). See Table 2 .

**Table 2:** Distribution of OHIP 14 responses to gender

Variable		OHIP 14 category			Total
		Low	Moderate	High	
Gender	Male	0(0%)	1(11.1%)	8(88.9%)	9
	Female	1(16.7%)	0(0%)	5(83.3%)	6
Total		1 (6.7%)	1 (6.7%)	13(86.7%)	15(100%)

Abbreviation: OHIP, Oral Health Impact Profile.

*p* -Value = 0.201.

Table 3 showed that there were six neglected patients, of which five patients were in a high OHIP 14 category (83.3%) and one patient was in a moderate category (16.7%). As for nonneglected, there were nine patients, of which eight patients were in a high OHIP category of 14 (88.9%) and one patient was in a moderate category (11.1%).

**Table 3:** Distribution of OHIP 14 response to neglected type

Variable		OHIP 14 category			Total
		Low	Moderate	High	
Neglected type	Neglected	0(0%)	1(16,7%)	5(83.3%)	6
	Nonneglected	1(11.1%)	0(0%)	8(88.9%)	9
Total		1 (6.7%)	1(6.7%)	13(86.7%)	15(100%)

Abbreviation: OHIP, Oral Health Impact Profile.

*p-* Value = 0.080.

There were seven patients with closed reduction, of which five patients were in a high OHIP 14 category (71.4%), one patient was in a moderate category (14.3%), and one patient was in a low category (14.3%). Meanwhile, for patients with open reduction, there were eight patients, of which eight patients were in a high OHIP 14 category (100%). See Table 4 .

**Table 4:** Distribution of OHIP 14 responses to types of management

Variable		OHIP 14 category			Total
		Low	Moderate	High	
Type of management	Closed reduction	1(14.3%)	1(14.3%)	5(71.4%)	7
	Open reduction	0 (0%)	0(0%)	8(100%)	8
Total		1 (6.7%)	1(6.7%)	13(86.7%)	15(100%)

Abbreviation: OHIP, Oral Health Impact Profile.

*p* -Value = 0.419.

### Response Distribution Based on General Oral Health Assessment Index

At the age of 18 to 40 years, there were 13 patients, of which 1 patient was in a high category of GOHAI (7.7%), 6 patients were in a moderate category (46.2%), and 6 patients were in a low category (46.2%). Meanwhile, in the age range of 41 to 65 years, there were two patients, and each patient was found to be in a moderate and low GOHAI category (50%). See Table 5 . There were nine male patients, one patient was in a high GOHAI category (11.1%), five patients were in the moderate category (55.6%), and three patients were in a low category (33.3%). As for female patients, there were six patients, of which two patients were in a moderate GOHAI category (33.3%) and there were four patients (66.7%) in a low category. See Table 6 .

**Table 5:** Distribution of GOHAI responses by age

Variable		GOHAI category			Total
		Low	Moderate	High	
Age	18–40 y	6(46.2%)	6(46.2%)	1(7.7%)	13
	41–65 y	1(50%)	1(50%)	0(0%)	2
Total		7 (46.7%)	7(46.7%)	1(6.7%)	15(100%)

Abbreviation: GOHAI, General Oral Health Assessment Index.

*p* -Value = 0.105.

**Table 6:** Distribution of GOHAI responses by gender

Variable		GOHAI category			Total
		Low	Moderate	High	
Gender	Male	3(33.3%)	5(55.6%)	1(11.1%)	9
	Female	4(66.7%)	2(33.3%)	0(0%)	6
Total		7(46.7%)	7 (46.7%)	1(11.1%)	15(100%)

Abbreviation: GOHAI, General Oral Health Assessment Index.

*p* -Value = 0.356.

There were six neglected patients, of which one patient was in a high category of GOHAI (16.7%), two patients in a moderate category (33.3%), and three patients in a low category (50%). Meanwhile, for nonneglected patients, there were nine patients, of which five patients were in a moderate GOHAI category (55.6%) and four patients in a low category (44.4%). See Table 7 .

**Table 7:** Distribution of GOHAI responses by type of neglected

Variable		GOHAI category			Total
		Low	Moderate	High	
Neglected type	Neglected	3(50.0%)	2(33.3%)	1(16.7%)	6
	Nonneglected	4(44.4%)	5(55.6%)	0(0%)	9
Total		7 (46.7%)	7(46.7%)	1(6.7%)	15(100%)

Abbreviation: GOHAI, General Oral Health Assessment Index.

*p* -Value = 0.356.

There were seven patients with closed reduction, where one patient was found to be in a high category of GOHAI (14.3%) and moderate and low categories each had three patients (42.9%). Meanwhile, for patients with open reduction, there were eight patients, of which four patients were in the moderate and low GOHAI categories (50%). See Table 8 .

**Table 8:** Distribution of GOHAI responses by management obtained

Variable		GOHAI category			Total
		Low	Moderate	High	
Management	Closed reduction	3(42.9%)	3(42.9%)	1(14.3%)	7
	Open reduction	4(50%)	4(50%)	0(0%)	8
Total		7 (46.7%)	7(46.7%)	1(6.7%)	15(100%)

Abbreviation: GOHAI, General Oral Health Assessment Index.

*p* -Value = 0.286.

## Discussion

The results of this study found that the number of patients with mandibular fractures comprised mostly young adults aged 18 to 40 years. This is because the age of 18 to 40 years is productive age with more activities than 41 to 65 years, so the risk of trauma is greater. This is in line with other studies which found that young adults are the age group with the highest incidence of mandibular fractures, such as China (4.1:1), 17 Korea (3:1), 18 Brazil (5.47:1), 19 America (4:1), 20 and England (6.6:1). 21

The results of this study also obtained data on the distribution of respondents for the category of fracture types that were dominated by condylar fractures, because anatomically the condyle region is the most fragile area and the area that gets the highest pressure when there is a collision in the anterior region of the mandible when compared with the other region that has the highest pressure. These results are in line with a study conducted by Natu et al who examined 102 cases of mandibular fracture and found that there were 29.1% condyle fractures, 24.5% angle fractures, 22% symphysis and parasymphysis fractures, 16% corpus fractures, and 3.1% fractures, dentoalveolar fractures, 1.7% ramus fractures, and 1.3% coronoid fractures. 22 While the most common fracture types were nonneglected, as many as nine cases with a percentage of 60%. This shows that most of the patients who come to the hospital are patients who have recently experienced trauma and receive immediate surgical treatment.

For the distribution of respondents based on their management, it was found that the majority of cases underwent surgery with open reduction, eight cases(53.33%). This is because most cases were displaced fractures. Another study also reported that mandibular fractures were dominated by condylar fractures with open reduction. Displaced mandibular fractures should be treated surgically and nondisplaced mandibular fractures conservatively. 14 In another study, it was also explained that the management of mandibular fractures with open reduction, in this case, ORIF, is the method of choice for surgery in patients with displaced fractures. 23 However, the results of this study did not find a significant difference in the quality of life of patients after surgery. Surgical management methods, both open reduction and closed reduction, did not have a significant effect on the results of the study. 24 According to the results of Omeje et al's study, in 56 cases with mandibular fractures, it was shown that patients receiving open reduction treatment experienced a decrease in quality of life due to pain after surgery, while those who were treated with closed reduction showed a decrease in quality of life due to physical and psychosocial discomfort. 25

From the evaluation of distribution data from respondents using the OHIP 14 parameter, it appears that the majority of respondents (86.7%) have a high quality of life level. The results of evaluation of the quality of life level using OHIP 14 based on age and sex showed that there was no significant difference in the level of quality of life with *p* -values of 0.154 and 0.080, respectively. The same was also shown in the distribution by fracture type, neglected type, and surgical management with *p* -values of 0.113, 0.080, and 0.419, respectively. These results indicate that based on the measurement of quality of life with parameter OHIP 14, there is no significant difference in the level of quality of life in each variable.

From the evaluation of distribution data from respondents using the GOHAI parameter, it appears that the majority of respondents (46.7%) have moderate and low levels of quality of life. The results of evaluation of quality of life level using GOHAI based on age and sex showed no significant difference with *p* -values of 0.105 and 0.356, respectively. The same was also observed in the distribution by fracture type, neglected type, and surgical management with *p* -values of 0.132, 0.356, and 0.286, respectively. These results indicate that based on the measurement of quality of life with GOHAI parameters, no significant difference was observed in the level of quality of life in each variable.

In the comparison of the patient's quality of life level between the OHIP 14 and GOHAI questionnaires, it was found that the distribution of respondents in the group with low, moderate, or high quality of life in the GOHAI category had a high level of quality of life based on the OHIP 14. This indicates that the standard of assessment on the GOHAI questionnaire is high. The GOHAI questionnaire has parameters that are considered more representative to measure the quality of life of patients than OHIP-14. 26

GOHAI gives a higher weight to measuring the quality of life by assessing functional limitations, pain, and discomfort, so the results obtained by GOHAI are more representative of oral disorders compared with those obtained by OHIP-14 which focuses on outcomes, such as psychological and behavioral outcomes which are less representative of quality of life after treatment. 27 28

## Conclusion

The results obtained from the distribution of data from respondents in this study found that the characteristics of age, gender, fracture type, neglected type, and management did not have a significant influence on the patient's quality of life after surgery, using both OHIP 14 and GOHAI questionnaires.

Patients with mandibular fractures who had surgical treatment according to the OHIP parameter 14 had a high quality of life on average among all respondents, while according to the GOHAI parameter, they had a moderate and low quality of life.
